# Hirayama Disease: A Case of an Albanian Woman Clinically Stabilized Without Surgery

**DOI:** 10.3389/fneur.2020.00183

**Published:** 2020-03-17

**Authors:** Annibale Antonioni, Mattia Fonderico, Enrico Granieri

**Affiliations:** ^1^Section of Neurology, Psychology and Psychiatry, Department of Biomedical and Specialty-Surgical Sciences, University of Ferrara, Ferrara, Italy; ^2^Department of Neuroscience, Firenze, Italy

**Keywords:** Hirayama disease, distal juvenile atrophy, cervical myelopathy, upper distal weakness, cervical flexion, hyposthenia

## Abstract

Hirayama Disease (HD) is a rare clinical condition that usually affects young people with preference for Asian males. It appears with unilateral distal amyotrophy or asymmetric bilateral amyotrophy of an upper limb which is to refer to an involvement of the spinal metamers C7-C8-T1. A clinical case of a female patient of Albanian nationality is described, with onset of the disease in adulthood and clinical and electrophysiological features suggestive of HD, without any characteristic imaging findings. Clinical investigations, EMG and radiological data facilitated the diagnosis and allowed the exclusion of degenerative forms of the motor neuron and radiculopathies. In this paper, we want to point out that the diagnosis of this pathology should be hypothesized even in the absence of characteristic epidemiological and imaging data.

## Introduction

Hirayama disease (HD), also called monomelic atrophy or distal juvenile muscular atrophy of the upper limbs, is a cervical myelopathy that generally has an insidious onset in adolescence and a slowly progressive and self-limiting course, with subsequent clinical stabilization in 3–5 years. HD seldom destabilizes and progresses after that period. Fujimori et al. (1), while sharing this prognostic definition of HD, also identified cases that can develop advanced forms of myelopathy with signs of a long tract, so they underlined the importance of long-term follow-up given the importance of a possible early surgery. It was first described in 1959 by Hirayama and is considered a rare disease, with <1,500 cases reported (2), especially in Asian countries. Usually, it occurs in males (3, 4) aged between 15 and 20 (5), but cases with a later onset (6) have also been reported in literature (7–9); so, it is important not to exclude the disease in patients with an age outside the typical range with compatible clinical examinations. The etiopathogenesis is unknown, but various hypotheses have been formulated: the most accredited one recognizes as a causal factor the bending of the neck, which involves displacement of the dural sac and, consequently, anterior crushing of spinal cord against the back of the vertebral bodies; this also explains the negative pressure that is generated in posterior venous plexus of the marrow (10) with compression of anterior epidural venous plexus and reduced drainage through the jugular veins (11). Maybe in males this mechanism is more frequent because, during puberty, the increase in stature causes a disproportion between the length of the spinal column and that of the spinal cord and dural sac (12), specially during bending. However, this explanation does not explain the reason for the asymmetry of involvement. Chen et al. (13) identify as a relevant factor that dura mater is a loose sheath. Shinomiya et al. (14) formulated another hypothesis regarding the role of flavo ligament in detachment of the dural sac (in women, often connective alterations correlate with abnormalities of the immune system, which is another factor considered in the genesis of HD). Biondi et al. (15) hypothesized that subclinical cervical traumas repeated during exercise were one cause of chronic alterations of the micro circulation; the group of Tashirho attributes a significant role to strenuous exercises of arms and repeated flexions of the neck (16). HD often occurs sporadically; however, Hirayama (17) reported family cases. The pathological anatomy shows an antero-posterior flattening of the spinal cord with atrophy of anterior horns, moderate gliosis without macrophage infiltration but with normal posterior horns and roots. It involves muscular weakness and atrophy—which runs obliquely along the volar and dorsal surfaces of the forearm—to the muscles of the hands and forearms, in most cases unilateral, but in 30% of patients an asymmetrical bilateral involvement may be present (11); it rarely occurs in symmetrical form (1). Tashiro and Pradhan suggested that “bilateral symmetrical Hirayama disease” is a more severe variant of HD (16, 18). Such involvements can cause limitations in hand movements and, consequently, difficulties in everyday activities. Furthermore, it's possible to find paresis from cold, fasciculations, tremor on fingers during extension with integrity of reflexes (with the exception of the radial and cubit-pronator stylus) and absence of pyramidal, sensory and cranial nerve signs. Cold extremities and sweating hands are frequent (19). Motor deficits are typically limited to C7, C8, and T1 myotomes, sometimes with involvement of the deltoid, biceps muscles and relative brachioradial sparing (16). Cases with atypical clinical manifestations have also been described: proximal limb involvement and positive signs of upper motor neuron disease (20); Holla et al. (21) reported the case of a patient who, in addition to a classical distal involvement, showed a significant involvement of the proximal peri-scapular muscles. There is no single test that can unequivocally confirm the diagnosis, which is based on medical history, clinical investigation, neuroimaging and EMG (22). Tashiro et al. (16) have recently proposed requirements for the diagnosis of DH:

1. Mainly distal weakness and muscular atrophy in the forearm and hand, although the limbs without apparent symptoms of HD may show electromyography abnormalities;
2. Almost always it involves the unilateral upper extremity;
3. Debut at the age between 10 and 20;
4. Insidious onset with gradual progression for the first few years, followed by stabilization;
5. No involvement of the lower limbs;
6. No sensory disturbance and tendon reflex abnormalities;
7. Exclusion of other diseases.

According to many studies, RM is the best diagnostic tool to confirm HD and exclude other spinal cord disorders (23) and it must be performed both in a neutral and a flexed position, comparing the two surveys. As reported by Lehman et al. (22), the most important findings that can be found are: loss of normal cervical lordosis; focal atrophy and asymmetric medullary flattening, best evaluated with T2-weighted TRM (with the “sign of the snake eyes” when the compression of the anterior horn of the marrow is highlighted with patient in maximum antero-flexion), anterior movement of posterior dural sac during neck flexion, prominence of the posterior epidural venous plexus. Electro physiological investigations show normal sensory conduction velocities, sensory nerve action potentials and terminal latencies (24). The magnitude of compound motor action potential may be decreased in affected and atrophied muscles (25). The differential diagnosis involves Amyotrophic Lateral Sclerosis, which can affect the systemic musculature and lead to respiratory failure, and Amyotrophic Cervical Spondylosis which causes damage to upper motor neurons (2). An early diagnosis, made complex by insidious onset and slow progression, is essential to establish an early treatment that decelerates the progression of the disease (12). For this purpose, the first choice is the use of a cervical collar that blocks the flexion of the neck for a period of about 3–4 years (even though patient compliance is generally low) (26, 27). Muscle-strengthening exercises and practices for the coordination of hand movements are also very useful. Vertebral fusion surgery (anterior, more effective and technically advantageous, or posterior) is reserved for advanced cases with persistent neurological deficit and clinical deterioration despite a correct conservative therapy, in order to merge the vertebrae involved in flexion lesion (28).

## Patient Presentation

### Clinical History

**Figure 1:**
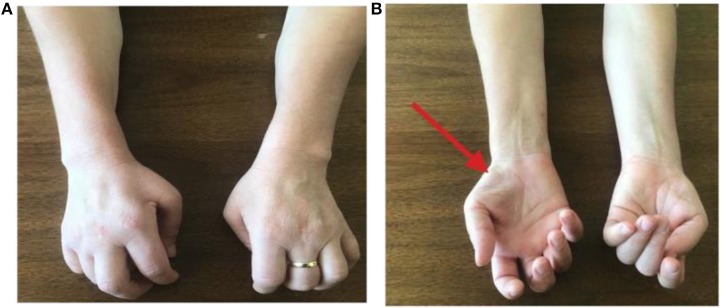
**(A,B)** Physical examination of the patient's upper limbs showed hypotrophy of the right tenar eminence, of the extensor muscles of the fingers bilaterally, of the common extensor muscles of the fingers, with thinning of the dorsal face of the forearm, more evident on the right. Furthermore, over time, a characteristic “claw posture” of hands has become evident, more on the left side than on the right, limiting daily activities.

MA is a 40-years-old Albanian woman born at the end of aeutocical birth. She has no family history of neurological diseases. Psycho-physical development was normal. She took her first steps at the age of 13 months and she began to pronounce the first words at 12 months. From the age of 14 she worked as an agricultural worker, in charge of milking cows and then she became a laundress. Normorhexica, normophagic, eupeptic. regular alvo, diuresis in the norm, and she does not report alterations of sleep. She began to manifest motor disorders, such as hyposthenia in manual work, at the age of twenty-eight. Since winter 2011, there has been a gradual difficulty in dorsal flexion of her wrists and extension of her fingers, especially on the left side at the beginning, then the disorder intensified and passed to the right. In addition, a negative thermal impact, swelling of fingers and worsening of motor symptoms after exposure to cold temperatures were also found. Over time, the symptoms have increased slightly, limiting an almost total manuality, further escalating local osteo-articular and muscle-tendon pains in her hands and, subsequently, in her wrists, elbows and shoulders. MA performed a negative cervical MRI in Albania. In 2011, she was subjected to neurological examinations, neurophysiological assessments, chest X-ray, cervical MRI (not performed in anteroflexion, perhaps because this pathology was not suspected) and blood tests in Italy. Neurological objectivity showed an exclusive motor deficit from bilateral C7-C8-T1 suffering, with a prevalent C7 involvement. The investigations highlighted: hypotrophy of the right tenar eminence, of the extensor muscles of the fingers bilaterally, of the common extensor muscles of the fingers, with thinning of the dorsal face of the forearm, more evident on the right (Figures 1A,B). The bicipital osteo-tendon reflexes are symmetrical and normo-evocable, radial stylus and pronator cubit absent on the left. No cranial and lower limb deficits have been detected. The neurophysiological studies of 2011 showed:

- Slight increase in slowing of sensory nerve conduction of the right median nerve on the left and the absence of anomalies of the remaining sensitive conduits examined in the superior limbs.
- Reduced cMAP amplitude from median stimulus on the right (response F absent), with prolonged distal motor latency bilaterally. The motor conduction of the right and left ulnar nerve was normal, with slight latency asymmetry of the right ulnar stimulus F response.
- EMG: signs of discrete chronic neurogenic restructuring, partly stabilized, in the muscles belonging to the C7-C8-T1 roots, bilateral but prevalent on the right.
- SEP (Somatosensory evoked potentials) (median nerve stimulus, tibialis posterior nerve stimulus, lumbar and cortical registration): normally all parameters.
- MEP (Motor Evoked Potentials) (transcranial and radicular magnetic stimulation, registration from abductor digiti minimi muscles): slight latency asymmetry of the MEP from cervical stimulus to the left, within normal limits the other parameters.
- MEP (transcranial and radicular magnetic stimulation, registration from tibialis anterior muscles): asymmetric post-MEP silent period, longer than normal to the left, within normal limits the other parameters.

A chest X-ray did not show a picture of supernumerary coast. Cervical RM was normal. Hematochimic tests showed positive ANA (1:320), which then regressed. IgGq1b were not investigated, but Baslo et al. showed the absence of correlation between their presence and HD (29). At the end of 2011, based on neurophysiological and neuroradiological data, the absence of a clear disorder of the motion neuron or a radicular suffering and in her clinical course, a condition like Hirayama's disease was diagnosed, affecting the spinal metamers C7-C8-T1. Symptomatic therapy with neurotrophic (alpha-lipoic acid 800 mg for 2 months) was suggested to the patient.

**Figure 2:**
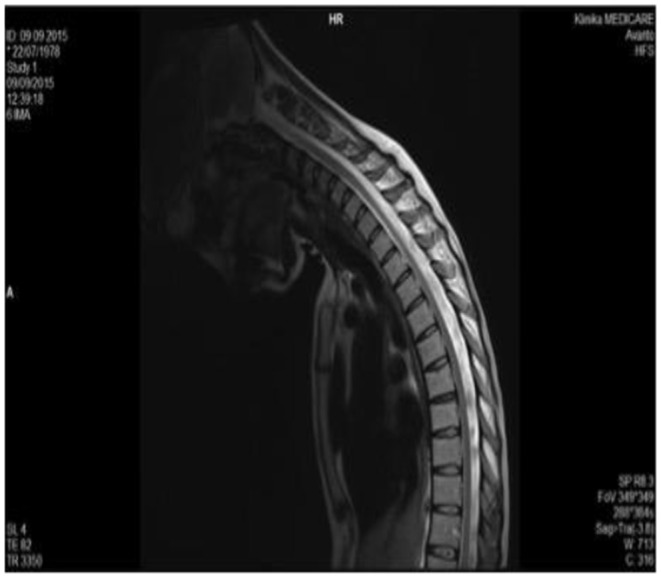
Cervical MRI of the neck in antero flexion, which does not show the hallmark of HD, i.e., the forward movement of the posterior wall of the inferior cervical dural sac (2015).

In 2015 cervical MRI was performed in Albania with projection in maximum antero-flexion of the neck, and it was normal (Figure 2).

**Figure 3:**
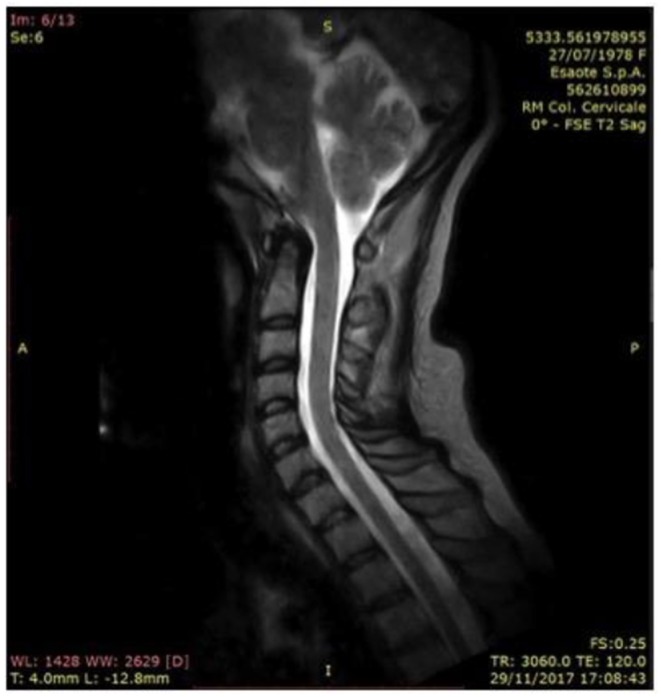
Vertebral column in hyperlordotic attitude, with fulcrum in C3-C4, where there are concomitant arthrosis of the posterior apophyseal; forward displacement of the spinal cord in the C1-C2 tract, which, in a neutral attitude of the neck in orthostatism, laps the posterior profile of the district ligamentous-meningeal component (2017).

In 2017, a further neurological examination and a MRI of the cervical spine were performed (Figure 3). Since the time of diagnosis, motor deficits have slightly evolved with motor limitations of the upper limbs, which lead to joint pains especially in her shoulders and hands. From the interview with the patient a constant need of help in carrying out daily activities emerged. The physical examination confirmed the findings described above, except for distal tactile hypoesthesia in fingers and hands. Furthermore, fatigue has emerged at the level of the scapula-humerus girdles during proximal movements. In the lower limbs a slight hyposthenia was found in the dorsiflexion of feet and toes, especially on the right, and pain on the front surface of her right thigh.

Every physical activity, even with her lower limbs, was performed with some limitation determined by joint pains distally and proximally. The MRI study of the cervical spine, performed in 2017, showed:

- Rachis caught in a hyperlordotic attitude;
- Vertebral species of normal amplitude;
- No pathological spinal cord inhomogeneity;
- Forward movement of the spinal cord in the C1-C2 tract, which in neutral position of the neck in standing position, touches the posterior profile of the district ligamentous-meningeal component and which in maximum active flexion causes spinal cord to such structures with obliteration of the liquor space. In maximal flexion acquisition, the spinal cord in the C4-C5 tract undergoes anterior displacement with extrinsic epidural compression.

The control EMG performed in Tirana in 2017 showed chronic suffering of the peripheral motor neuron, which was in the same condition as in the previous examination (2015). Therapy with methylprednisolone 16 mg was undertaken.

In addition to drug therapy, the use of a cervical collar was recommended, aimed at preventing neck flexion and slowing the progression of muscular atrophy. However, the use of this facility was suspended after a few months.

**Figure 4:**
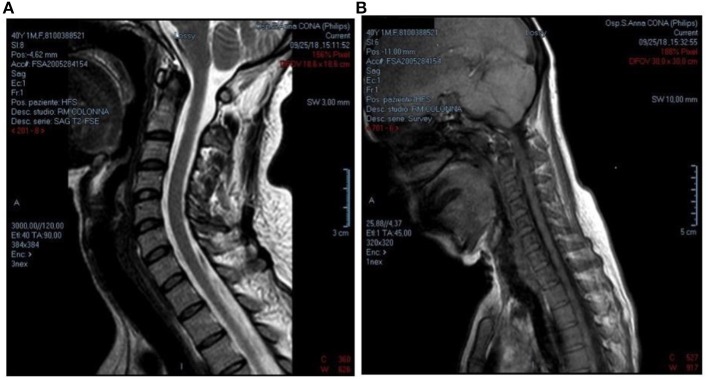
**(A,B)** RM of the cervical spine, performed in supine position, showed a spinal canal of normal morphology and amplitude, with a regular caliber in all its diameters and without intrinsic alterations in signal intensity. It was also found regular adherence of dural sac to the posterior surface of the vertebral arches. In anteroflexion of the neck, straightening of the cervical segment is observed without, however, signs of detachment of the dural sac from the internal surface of the vertebral arch (2018).

The physiokinesis therapy, recommended to the patient at the end of 2011, was very useful. The neurological examination carried out in March 2018 highlighted head and neck in a perennially lateral posture toward the left and intense pain evoked by the mobilization of the head or neck to the left, then improved at the physical examination in September 2018. Fatigability and pain remain in the scapulohumeral girdle during the execution of proximal movements and in the maintenance of the antigravity posture of Mingazzini. Compared to previous visits, the physical examination of the upper limbs showed an improvement in the motor functions of the hands, while the need for continuous help during daily activities remained. Even in the lower limbs, slight hyposthenia persists in the dorsiflexion of toes and feet, with limitation of physical activity. Muscle relaxant therapy with Tizanidine 2 mg and Duloxetine 30 mg combined with physiotherapy exercises led to a slight improvement in the clinical picture. The RM of the cervical spine performed at the end of September 2018 in supine position showed a spinal canal of normal morphology and amplitude (Figures 4A,B). The adherence of the dural sac to the posterior surface of the vertebral arches was also found to be regular. In anteroflexion of the neck the straightening of the cervical segment was observed, without however visible signs of any detachment of the dural sac. The study of MEP performed in September 2018, showed similar results to previous investigations.

Through the last EMG of lower limbs, rectus femoral, gastrocnemic, sural, peroneal, vastus medialis and anterior tibial muscles were examined and a neurogenic damage to the middle-lower roots of the right lumbosacral plexus was found (in particular, at the level of the muscles tibialis anterior, gastrocnemic and vastus medialis); in order to confirm the diagnostic hypothesis of disc protrusion, a CT scan of the lumbosacral spine was performed in Albania. It highlighted the presence of moderate scoliosis associated with chronic intervertebral disc disease at the L4-L5 level; in this location, a right hernial protrusion, present for a long time, has resulted in narrowing of the lateral foramen and chronic sensory-motor radicular damage of L5, congruent with the strength deficit described and with the result of the EMG test. Blood chemistry tests were repeated in January 2020 in order to look for the presence of markers of inflammation and auto-immune pathologies, with negative results.

### Neuroimaging

RM 2015, Figure 2.
RM 2017, Figure 3.
RM 2018, Figures 4A,B (in flexion).

## Discussion and Conclusion

This case of MA has peculiar epidemiological characteristics: it concerns a Caucasian woman, of Albanian origin, in whom the onset of the symptomatology occurred at the age of 28, later than the average age described in literature.

An important anamnestic data is the work carried out by the patient at a young age: laundress and agricultural worker, which involved a frequent attitude of antero-flexion of the neck and laborious movements of the arms: these hard jobs may have played a role in the pathogenesis of the disease. The patient's clinical condition is typical of HD. Muscular alteration has asymmetric bilateral topography, with greater involvement of the right upper limb. The patient's hands at present have a characteristic “claw posture,” more on the left side than on the right, limiting daily activities. Other typical signs are the swelling of fingers which are generally cold, cold paresis and lack of disturbances and pyramidal signs.

Repeated neurological examinations, from 2011 to 2016, showed a slow evolution of motor deficits, with further limitations of movement and progressive reductions of the primary activities in daily life, up to a period of stabilization. Overall the pathology showed a slowly progressive course, from the age of 28 to 36 years when a subsequent stabilization of the symptoms started (2017).

After this period of stabilization, a minimal involvement of the lower limb took place, mainly on the right side, with slight hyposthenia in the dorsiflexion of the foot and toes and with small limitations of her physical activity; this framework is compatible with L4-L5 radiculopathy on the right (as confirmed by the EMG and MRI). The neurophysiological investigations carried out confirmed the presence of chronic neurogenic muscle suffering from the muscles belonging to the lower middle roots of the brachial plexus, bilateral mainly on the right, consistent with a metameric spinal disease involving C7-C8- T1. SEP and MEP were substantially normal, just a very small worsening of slight anomalies of the conduction of the central right pathway. MRI has proven to be the best diagnostic tool.

However, cervical MRI images did not show typical findings. One of the most significant MRI imaging findings is the forward movement of the dural sac, however, this sign is not always present in HD: Zhou et al. (3) reported a prevalence of 71%.

Therefore, even in the case of MA, despite the absence of significant imaging findings, the clinical features and electrophysiological results are consistent with the diagnosis of HD. In this case, the neurophysiological results also provided important information for the differential diagnosis. EMG findings confirmed neurogenic muscle suffering, excluding a distal myelopathy. Clinical signs of involvement of the first motor neuron, sensitivity changes, ataxia, extra-pyramidal signs, or changes in cranial nerve function were not appreciated. All this was sufficient to rule out disorders of the neuron of motion or neurodegenerative disorders.

In the present case, the treatment was conservative, but a cervical collar was used only for a short time. The slight improvement is due to muscle relaxants and daily physiotherapy exercises of hands. The hand exercises, such as flexion, bending, abduction, crossing of fingers against resistance and adduction of the little finger toward the base of the thumb, have led to a slight improvement in the movements of the right hand, however it was not sufficient for greater autonomy in activities of daily life. The left hand showed no significant improvement and a claw-like posture remains. Despite lower limb exercises, such as hip extensions or thigh rotations, flexion and pronation of foot, hyposthenia remains in dorsiflexion.

In conclusion, HD should be considered in any patient presenting a history of hyposthenia and amyotrophy of the upper limbs and hands, with an insidious onset of symptoms and a slowly progressive course. The most frequent symptoms are hyposthenia, cold paresis, atrophy, and sometimes there may be tremors, without sensory, pyramidal or cranial nerve disorders. The subjects who are most affected are adolescent males of Asian ethnicity, but there are cases outside the typical ranges. We report the case of an Albanian woman with onset of symptoms at a more advanced age than normal. Moreover, atypically, she shows an asymmetric involvement of her upper limbs. In this case, clinical and diagnostic follow-up documented a stabilization of the pathological condition. Based on the anamnesis (work with head bowed forward and repeated efforts in the upper limbs, a known risk factor reported in literature), clinical and neurophysiological tests, the likely hypothesis is that it is a case of HD, which is compatible for the data reported. However, the presence of negative imaging (fundamental diagnostic element) prevents the confirmation of the diagnosis with certainty and leads to consider even other pathologies belonging to the group of motor neuron diseases.

## Data Availability Statement

The datasets generated for this study are available on request to the corresponding author.

## Ethics Statement

Written informed consent was obtained from the individual(s) for the publication of any potentially identifiable images or data included in this article.

## Author Contributions

EG made the first supposition about the disease of the patient and has coordinated the diagnostic and clinical iter. AA and MF have followed the clinical pathway of the patient and collected the patient's data.

### Conflict of Interest

The authors declare that the research was conducted in the absence of any commercial or financial relationships that could be construed as a potential conflict of interest.
